# Experience of the surgeon affects the success rate of the placement of orthodontic miniplates

**DOI:** 10.2340/aos.v84.43123

**Published:** 2025-03-11

**Authors:** Elina Savolainen, Lotta Veistinen, Antti Asikainen, Anu Kiukkonen

**Affiliations:** a Dental Care, Health and Social Services, City of Helsinki, Helsinki, Finland; b Department of Oral and Maxillofacial Diseases, Head and Neck Center, University of Helsinki and Helsinki University Hospital, Helsinki, Finland

**Keywords:** BAMP, resident, skeletal anchorage, orthodontics, surgeon

## Abstract

**Background:**

Skeletal Class III patients can be treated with bone anchored maxillary protraction (BAMP) treatment. The placement of miniplates is an invasive operation and needs to be carried out under general anesthesia.

**Aim:**

The aim of this retrospective study was to determine the failure rate of miniplates.

**Methods:**

All the patients who had miniplates placed for BAMP treatment between January 2010 and April 2020 in Department of Oral and Maxillofacial Diseases, Helsinki University Hospital, Finland were included in this study. The patient records were retrospectively screened. The success rate of the placement of orthodontic miniplates was evaluated between surgical residents (Group 1) and consultant surgeons (Group 2).

**Materials:**

The study group consisted of 164 miniplates in 42 patients. The miniplates were divided into two groups, depending on the placement operator. Group 1 consisted of 86 miniplates placed by surgical residents. Group 2 consisted of 78 miniplates placed by consultant surgeons.

**Results:**

In Group 1 (11 females, 14 males) and Group 2 (16 females, 9 males). The overall failure rate of miniplates was 23.8% (*n* = 39). The failure rate for surgical residents (Group 1) was 31.4% (*n* = 27) and for consultant surgeons (Group 2), 15.3% (*n* = 12) (*p* < 0.05). Out of all the failed miniplates 69.2% (*n* = 27) were placed by residents and 30.8% (*n* = 12) by consultants.

**Limitations:**

This retrospective study consisted of a limited number of patients.

**Conclusion:**

The failure rate of miniplates placed by consultant surgeons was lower compared to surgical residents (*p* < 0.05).

## Introduction

Skeletal Class III malocclusion is caused by a discrepancy between the mandible and the maxilla, in which the growth of the maxilla has been deficient, the growth of the mandible has been excessive, or both [1]. This usually causes an anterior crossbite and a concave profile. Class III malocclusion lowers the patient’s quality of life due to masticatory problems, incisal wear, and an unaesthetic facial appearance [2, 3]. The prevalence of Class III malocclusion varies greatly among populations [4]. It is rare in Finland, affecting approximately 1% of the Finnish population [5]. Both environmental and genetic factors influence the development of Class III malocclusion [6, 7].

The treatment window for skeletal Class III malocclusion during active growth is narrow and the biggest factor for long-term stability regarding the treatment outcome is the direction and the amount of residual growth of the mandible and the maxilla [8]. Earlier studies suggest that it is possible to accelerate the forward growth of the maxilla with treatment, but it is difficult to restrict the growth of the mandible [9]. Traditional treatment protocols are facemask therapy with rapid maxillary expansion appliance (FM-RME), chin-cup treatment and orthognathic surgery. The treatment of Class III malocclusion during growth with bone-anchored maxillary protraction (BAMP), where intermaxillary elastics are placed on miniplates between the upper and lower jaws has widely increased in recent years. The objective is to attain skeletal maxillary advancement and to avoid dentoalveolar compensation. The intermaxillary traction can be applied for 24 h a day and patient compliance might be needed less than with the FM-RME treatment [10]. With both the BAMP treatment and FM-RME therapy, skeletal changes can be attained [9–11], but there is a lack of research on long-term stability regarding the treatment results with the BAMP treatment. The miniplates are recommended for patients aged 11 years and older. The failure rate of miniplates is higher in patients under 11 years of age [12] In literature, the success rates for miniplates vary between 92 and 99% [13–18]. The placement of miniplates is an invasive procedure that requires opening of a mucoperiosteal flap and the procedure is done by oral surgeons, usually under general anesthesia in children. The miniplates are placed in the maxilla buccally in the zygomatic buttress and in the mandible labially between the canines and lateral incisors and they are attached to the bone by using mini-screws.

The Finnish public healthcare system and the public dental service (PDS) offer publicly funded orthodontic treatments for all children under 18 years, who are diagnosed with severe malocclusion, including Class III malocclusion with an anterior crossbite. In Finland the BAMP treatment has been in use for a little over a decade. Nowadays, Helsinki University Hospital (HUH) is mainly responsible for the placement of miniplates for Class III patients in the Uusimaa region consisting of 1.8 million inhabitants. HUH is a teaching hospital, where consultant surgeons and surgical residents are responsible for the placement of the miniplates. In Finland, children who are diagnosed with anterior crossbite are routinely first treated with FM-RME. BAMP treatment therefore often serves as a second phase treatment in patients who have not responded to FM-RME treatment favourably. The method of choice for the treatment of Class III malocclusion should be effective and stable in the long term, as well as cost-effective, and the burden of care should not be excessive for the patient.

The aim of this retrospective study was to determine the failure rate of miniplates and evaluate the influence of the surgeon’s experience on the failure rate.

## Materials and methods

### Subjects

Patients’ records from cases using miniplates for BAMP treatment at the Department of Oral and Maxillofacial Diseases, Head and Neck Center, HUH were retrospectively studied. Specialist orthodontists have made referrals for the insertion of the plates for patients who would benefit from BAMP treatment. In borderline cases patients were examined by specialist orthodontist and a surgeon prior to the placement of the plates and the need for BAMP treatment was re-evaluated in HUH. All the patients with miniplates placed from January 2010 to April 2020 were included in this study. Patients with missing patient records for the insertion or the extraction of the miniplates were excluded from our study (*n* = 5). The study group consisted of 164 miniplates in 42 patients (Table 1). The miniplates were divided into two groups depending on the placement operator. Patient’s medical history is presented in Table 3. In Group 1 (Table 2), the miniplates were placed by the resident surgeons, whereas in Group 2, the miniplates were placed by the consultant surgeons.

**Table 1:** Number of miniplates placed, patients, and the mean ages at the beginning of the treatment at HUH between January 2010 till April 2020.

Patient information and miniplate placement overview	All/failed	Odds ratio (95% confidence level)
Mean age of the patients (years)	12.2 ± 1.0/11.9 ± 1.1	
Number of patients	42/16 (38.1%)	
Female	22/6 (27.3%)	
Male	20/10 (50%)	
Failed Female/male (*p*-value)	*p* = 0.389	0.375 (0.104 1.354)
Miniplates placed	164/39 (23.8%)	
Place of miniplates		
Maxillary plates	82/22 (26.8%)	
Right side	41/10 (2..4%)	
Left side	41/12 (29.2%)	
Failed right/left side (*p*-value)	*p* = 0.618	0.780 (0.293 2.077)
Mandibular plates	82/17 (20.7%)	
Right side	41/9 (22..0%)	
Left side	41/8 (19.5%)	
Failed right/left side (*p*-value)	*p* = 0.714	1.198 (0.410 3.495)
Failed Maxillary/Mandibular plates (*p*-value)	*p* = 0.688	1.402 (0.68 2.891)
Number of screws per plate		
2 screws	98/16 (16.3%)	
Plate placed in maxilla		
With 2 screws	33/5 (15.2%)	
Right side	18/3 (16.7%)	
Left side	15/2 (13.3%)	
Failed right/left side (*p*-value)	*p* = 0.413	2.500 (0.409 15.293)
With 3 screws	37/11 (29.7%)	
Right side	17/4 (23.5%)	
Left side	20/7 (35%)	
Failed Right/left side (*p*-value)	*p* = 0.495	0.571 (0.134 2.44)
Failed with 3 screws/2 screws (*p*-value)	*p* = 0.147	2.369 (0.725 7.743)
Plate placed in mandible		
With 2 screws	65/11 (16.9%)	
Right side	32/6 (18.8%	
Left side	33/5 (15.2%)	
Failed right/left (*p*-value)	*p* = 0.714	1.292 (0.352 4.748)
No information	26/12 (46.2%)	

**Table 2:** Miniplates placed divided into Groups; Group 1 consists of the plates placed by residents, and Group 2 consists of the plates placed by consultants. (Values with *p* ≤.05 are marked *.)

Patient information and miniplate placement overview	Group 1/failed	Group 2/failed
Mean age of the patient at the beginning of the treatment (years)	12.4 ± 1.1/12.1 ± 1.0	12.2 ± 1.1/11.5 ± 0.5
Miniplates placed	86/27 (31.4%)	78/12 (15.3%)
Group 1/Group 2 Failed (*p*-value)	27/12 (*p =* 0.016*)	
Odds Ratio (95% confidence level)	2.517 (1.171 5.411)	
Number of patients	25/11 (44%)	25/6 (24%)
Female	11/4 (36.4%)	16/3 (18.6%)
Male	14/7 (50%)	9/3 (33.3%)
Failed Female/male (*p*-value)	*p* = 0.689	*p* = 0.642
Odds Ratio (95% confidence level)	0.571 (0.114 2.872)	0.462 (0.71 2.994)
Plates placed, divided by the experience of the resident		
1–2 year	46/11 (23.9%)	
3–4 year	32/14 (43.8%)	
5–6 year	8/2 (25%)	
Failed (1.-2.)/(3.-4.)/(5.-6.) (*p*-value)	*p* = 0.102	
Place of miniplates		
Maxillary plates	38/15 (39.5%)	44/7 (15.9%)
Right side	19/8 (42.1%)	22/3 (13.6%)
Left side	19/7 (36.8%)	22/4 (18.2%)
Failed Right/Left side (*p*-value)	*p* = 0.740	*p* = 1.000
Odds Ratio (95% confidence level)	0.802 (0.218 2.952)	0.711 (0.139 3.626)
Mandibular plates	48/12 (25%)	38/5 (13.2%)
Right Side	24/6 (25%)	19/3 (15.8%)
Left side	24/6 (25%)	19/2 (10.5%)
Failed Right/left side (*p*-value)	*p* = 1.000	*p* = 1.000
Odds Ratio (95% confidence level)	1.000 (0.271 3.694)	1.594 (0.235 10.817)
Failed maxillary/mandibular plates (*p*-value)	*p* = 0.151	*p* = 0.725
Odds Ratio (95% confidence level)	1.957 (0.778 4.919)	1.249 (0.361 4.314)
Plates attached by number of screws		
With 2 screws in maxilla and mandible	49/12 (24.5%)	49/4 (8.2%)
Plate placed in maxilla		
With 2 screws	12/4 (33.3%)	21/1 (4.8%)
Right side	4/3 (75%)	10/0 (0%)
Left side	8/1 (12.5%)	11/1 (9.1%)
Failed Right/left side (*p*-value)	*p* = 0.067	*p* = 1.000
Odds Ratio (95% confidence level)	21.000 (0.961 458.842)	1.100 (0.913 1.326)
With 3 screws	20/7 (35%)	17/4 (23.5%)
Right side	8/5/62.5%)	9/2 (22.2%)
Left side	12/2 (16.7%)	8/2 (25%)
Failed Right/left side (*p*-value)	*p* = 0.062	*p* = 1.000
Odds Ratio (95% confidence level)	8.333 (1.034 67.142)	0.857 (0.091 8.075)
Failed with 3 screws/2 screws (*p*-value)	*p* = 1.000	*p* = 0.152
Odds Ratio (95% confidence level)	0.929 (0.2054 0.2110)	0.163 (0.016 1.621)
Plate placed in mandible		
With 2 screws	37/8 (21.6%)	28/3 (10.7%)
Right side	18/4 (22.2%)	14/2 (14.3%)
Left side	19/4 (21.1%)	14/1 (7.1%)
Failed Right/left (*p*-value)	*p* = 1.0	*p* = 1.000
Odds Ratio (95% confidence level)	1.071 (0.224 5.128)	2.167 (0.173 27.075)
No information on the amount of screws	15/8 (53.3%)	12/4 (33.3%)

**Table 3:** Medical history of the patients.

Medical history	No. of patients (*n* = 42)(%)	Group 1 (*n* = 25) (%)	Group 2 (*n* = 25) (%)
Healthy	29 (69)	19 (76)	15 (60)
Autoimmune disease (asthma, celiac disease, etc.)	11 (26)	4 (16)	7 (28)
Syndromes	2 (5)	1 (4)	1 (4)
Allergies	5 (12)	0	5 (20)

### Surgical methods and placing sites

The miniplates were placed in the maxilla in the zygomatic buttress (*n* = 82) and in the mandible (*n* = 82) between the canines and lateral incisors or between the canine and the first premolar depending on the space between the roots. All the miniplates were placed under general anesthesia by flap surgery as previously described by De Clerck et al. [19], performed by a consultant oral surgeon or a resident in HUH. The written instructions for the placement of the miniplates were provided for the residents. A video presentation of the placement of the miniplates was provided for the residents prior to the first surgery. Residents were supervised intraoperatively. Residents first follow the senior consultant placing a miniplate on one side, before placing a miniplate on the other side of a patient. During the training, the residents will place miniplates on both sides of the patient. Postsurgical instructions were given to the patients. Patients were instructed not to manipulate the miniplates with fingers, and orthodontic wax was given for the possible irritation of the oral mucosa. Maxillomandibular elastics were placed between the upper and the lower miniplate on each side 2 weeks after the operation at the earliest.

Two different types of miniplates were used in this study; Bollard modified miniplate, Tita-Link (*n* = 160) and DePuy Synthes miniplate (*n* = 4). Loose miniplates that needed to be replaced or removed were considered failed cases.

### Methods

All data variables were collected from the patients’ records by a single researcher using a pre-defined data collection sheet in Microsoft Excel (Microsoft Excel, version 16, Seattle, USA).

### Statistical analysis

The statistical analyses were carried out by using IBM SPSS Statistics, version 27. The chi-square analysis and Fisher’s exact test were used to investigate whether there was a significant association between the failure of the miniplates and the gender of the patient or the placement site, or between the placement by consultants versus resident oral surgeons. To avoid pseudoreplication we included only one miniplate per placement site in analysis of the failure rate and only one miniplate per person in analysis comparing the association between genders. All *p*-values under 0.05 were considered significant. Survival analysis was conducted for visualization.

### Ethical approval

The protocol of this retrospective study was approved by the Hospital District of Helsinki and Uusimaa (HUS/126/2021). Principles outlined in the Declaration of Helsinki were followed.

## Results

Out of the 164 miniplates placed, 39 failed in 15 patients, six females and nine males (Table 1). The overall failure rate was 23.8%. There was no statistically significant difference between the failure rate of female and male patients (*p* = 0.389). Out of the 39 failed miniplates, 22 were placed in the maxilla and 17 in the mandible. There was no statistically significant difference between the failure rate of the miniplates placed in the maxilla versus the mandible (*p* = 0.688) or between the right or left side of the jaw. The mean age for the failed patients was 11.9 ± 1.1 years. Six patients lost only one miniplate, two patients lost two miniplates, one patient lost three miniplates, and six patients lost all four miniplates. A total of 60% of the patients who had a miniplate failure lost two or more miniplates. Two patients showed repeated failures, one plate per patient failed two times, both patients’ plates were placed in the left side of the mandible. Other complications reported after the procedure are listed in Table 4.

**Table 4:** All complications with miniplates per patient.

Complications	All (*n* = 42)	Group 1 (*n* = 25)	Group 2 (*n* = 25)
Failure	25 (60%)	11 (44%)	6 (24%)
No complications	23 (55%)	12 (48%)	18 (72%)
Pain	2	1	1
Ulceration	2	1	1
Inflammation	5	2	3
Broken plate(hook)	1	1	0
Patient compliance	2	1	1
Plate mobility	2	2	0
Soft tissue hyperplasia	2	2	0
Hook to soft tissue	3	3	1

### Group 1

Group 1 consisted of 86 miniplates in 25 patients, 14 males and 11 females, with a mean age of 12.4 ±1.1 years. In Group 1, the main orthodontic diagnosis was maxillary retrognathia and hypoplasia in 15 (60%) patients, mandibular prognathia and macrognathia in seven (28%) patients and a cross bite in two (8%) patients. In Group 1 27 miniplates failed in 11 patients, four females and seven males, with a failure rate of 31.4% (Table 2). There was no statistically significant difference between the failure rate of female and male patients (*p* = 0.689). Out of the 27 failed miniplates, 15 were placed in the maxilla and 12 in the mandible. There was no statistically significant difference between the failure rate of miniplates placed in the maxilla versus the mandible (*p* = 0.151). There were no statistically significant differences in the failure rates between 1st and 2nd year, 3rd and 4th year or 5th and 6th year residents (*p* = 0.102). The mean age of the failed patients was 12.1±1.0 years.

**Figure 1:**
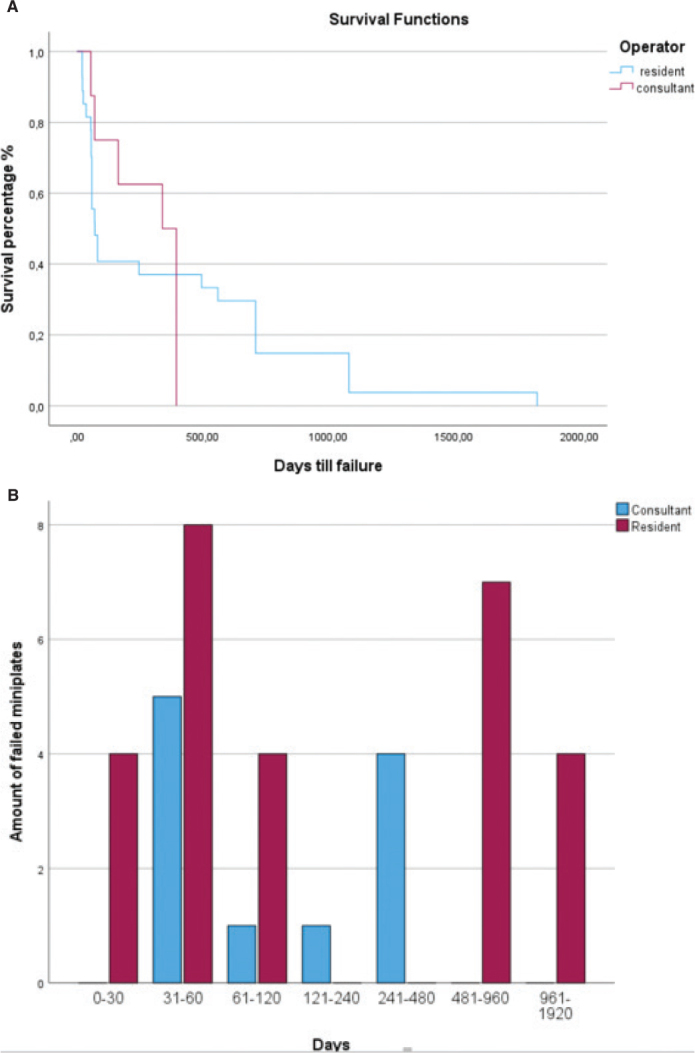
(A) Survival analysis of all the failed plates placed by residents (blue) and consultant oral surgeons (red). (B) Cluster Chart.

Only four miniplates failed within the first 30 days, and within the first 82 days, 16 (59.3%) miniplates failed. The mean duration for the treatment was 1,222 days (Figure 1).

### Group 2

Group 2 consisted of 78 miniplates in 25 patients, nine males and 16 females, with a mean age of 12.2±1.1 years. In Group 2, the main orthodontic diagnosis was maxillary retrognathia and hypoplasia in 14 (56%) patients, mandibular prognathia and macrognathia in four (16%) patients, and a cross bite in four (16%) patients.

In Group 2, 12 miniplates failed in six patients, three female and three males, with a failure rate of 15.3%. There was no statistically significant difference between the failure rate of female and male patients (*p* = 0.642). Seven of the failed miniplates were placed in the maxilla and five in the mandible. There was no statistically significant difference between the failure rate of miniplates placed in the maxilla versus the mandible (*p* = 0.884). The mean age of the failed patients was 11.5±0.5 years.

Zero miniplates failed within the first 30 days and 6 (50%) of the miniplates failed within the first 70 days; the mean duration for the treatment was 1,153 days (Figure 1). A total of 69.2% of all of the miniplate failures (27/39) occurred in Group 1. There was a statistically significant difference between the failure rate for miniplates placed by surgical residents versus consultants (*p* = 0.016) OR 2.517 95% CL (1.171 5.411).

## Discussion

The aim of this study was to determine the failure rate for miniplates placed for BAMP treatment in a publicly funded teaching hospital and to evaluate if there is a difference between the failure rate of miniplates placed by residents and consultant surgeons. The placement of miniplates is an invasive procedure that usually requires general anesthesia in children and patients experience post-operative discomfort [16]. The treatment of any malocclusion should be effective, and the complications of treatment should be avoided. It is also important that orthodontists and surgeons consider how much the treatment increases the burden of care for the patient [20]. In publicly funded healthcare it is necessary to consider the cost-effectiveness of the treatment. The low success rate in our study is not in line with the success rates reported in the literature. Other studies have reported 92–99% success rates for miniplates [13, 14, 16, 18, 21]. In many studies only a handful of experienced surgeons placed the miniplates [13, 18, 21–23]. However, in this study the failure rate was significantly higher for miniplates placed by surgical residents than consultant surgeons. This could suggest that the experience of the surgeon lowers the failure rate significantly. In other fields of medicine previous research has shown that the residents’ participation as the co-surgeon in the operation did not impact patient outcomes [24], nor did the patient outcomes differ between the surgeries performed by residents and surgeons [25]. Miniplates and screws will not osseointegrate like dental implants, although the bone grows on to the screws and over the miniplates with time. The miniplates are held in place by the mechanical stability of the screws. The lesser the trauma takes place during implantation, the better the ingrowth of new bone and stability of the plate will be [26]. Both self-tapping and -drilling screws were used. Especially with self-drilling screws, care must be taken that the screwing process will not oscillate to compromise the stability of the screw. This might be one reason why there was a significant difference in loosening the miniplates placed by the residents. Also, care must be taken in bending the miniplates according to the manufacturer’s instructions. In this study, it was not possible to assess if the miniplates were perforating the attached gingiva optimally and if the used force vector was associated with hardware failure, as well as considering the bone quality and the amount. In this study, it was not possible to evaluate how the thickness or quality of the bone affects the stability of miniplates.

Since the failure rate of miniplates placed by residents was significantly higher compared to consultant surgeons, it should be considered carefully if this treatment method is safe to use at a teaching hospital. On the other hand, it is important to educate residents to place miniplates correctly, if the BAMP treatment is an effective method to treat Class III malocclusions. At present, the BAMP treatment outcome is controversial [27] and there are no long-term follow up results available. It is crucial that the teaching is done under careful supervision to reduce the failure rate of miniplates in order to lighten the burden of care for the patients. The small study group (*n* = 42) and retrospective research setting are limitations in deriving unequivocal conclusions from the results.

Future research is needed with more substantial material in Finland to evaluate the treatment outcomes, the failure rate of miniplates, and the occlusal stability of BAMP treated patients. Since the failure rate is proven to be higher in this study compared to the literature, it is important to carefully supervise the residents during the placement of miniplates.

## Conclusions

The failure rate was significantly higher for the miniplates placed by residents than consultant surgeons, suggesting that the experience of the surgeon lowers the failure rate significantly. Therefore, in order to reduce the failure rate, it is required to carefully supervise the surgical residents during the placement of miniplates.

## Data Availability

The data underlying this article will be shared on reasonable request to the corresponding author.
